# The Association of Handgrip Strength and Type 2 Diabetes Mellitus in Six Ethnic Groups: An Analysis of the HELIUS Study

**DOI:** 10.1371/journal.pone.0137739

**Published:** 2015-09-14

**Authors:** Anne-Lotte L. F. van der Kooi, Marieke B. Snijder, Ron J. G. Peters, Irene G. M. van Valkengoed

**Affiliations:** 1 Department of Public Health, Academic Medical Center of the University of Amsterdam, Amsterdam, the Netherlands; 2 Department of Cardiology, Academic Medical Center of the University of Amsterdam, Amsterdam, the Netherlands; National Cancer Center, JAPAN

## Abstract

We investigated whether ethnic differences in handgrip strength, a marker of poor muscle strength and quality, is a potential cause of ethnic disparities in type 2 diabetes mellitus. We included 2086 Dutch, 2216 South Asian Surinamese, 2084 African Surinamese, 1786 Ghanaian, 2223 Turkish and 2199 Moroccan origin participants from the HELIUS study. We analyzed ethnic differences in handgrip strength, and its association with type 2 diabetes mellitus using logistic regression analyses adjusted for socio-demographic factors, body composition and lifestyle factors. Additionally, we investigated whether handgrip strength explained the ethnic differences in type 2 diabetes mellitus. We found that handgrip strength differed significantly across ethnic groups. After full adjustment, we found an inverse association with type 2 diabetes mellitus (OR 0.95; 95% CI 0.92–0.97) that did not differ substantially between ethnic groups, men and among women, and lean and overweight individuals. The association was not affected by the measure used to define type 2 diabetes mellitus, but was attenuated by exclusion of people with known diabetes. The ethnic differences in type 2 diabetes mellitus were not explained by handgrip strength (e.g. the OR for the South Asian Surinamese vs. Dutch changed from 5.03; 3.69–6.68 to 4.87; 3.57–6.65). In conclusion, we found large ethnic differences in handgrip strength and a consistent association of low handgrip strength with prevalent type 2 diabetes mellitus. This suggests that handgrip strength may be investigated as a target for intervention or a marker to identify people at risk of type 2 diabetes mellitus.

## Introduction

Given the association of type 2 diabetes mellitus with high morbidity and mortality, the persistent expansion of the type 2 diabetes mellitus epidemic is a major public health challenge [1–4]. Studies show that specific ethnic groups are disproportionally affected by type 2 diabetes mellitus even when exposed to similar environmental challenges [5–8]. For instance, in a study in the Netherlands, the prevalence of type 2 diabetes mellitus in South-Asian Surinamese, African Surinamese and Dutch origin participants was 25.6%, 12.7% and 6.8% respectively [8]. These ethnic differences can only in part be explained by conventional risk factors, such as obesity. Therefore, other risk factors may contribute to the complex development of type 2 diabetes mellitus and could possibly support the early detection and prevention of the disease.

Previous studies have shown that handgrip strength, a marker of muscle strength and quality is a risk factor for mortality due to cardiovascular disease, type 2 diabetes mellitus and other diseases [9–15]. In addition, studies have demonstrated an association between muscle mass and muscle function and the glucose metabolism [16–18]. Resistance exercise training to increase muscle mass and improve muscle function has been shown to enhance insulin action in skeletal muscle, improve glucose tolerance, and decrease glycated hemoglobin concentrations [16].

However, as has recently been stressed by others [15], little evidence on the association of handgrip strength with type 2 diabetes mellitus is available from populations with varying ethnic backgrounds in different settings within one region or country. The available evidence is mainly from North American studies on Hispanic, African Americans or American Japanese populations [11, 12, 14, 19]. Evidence on the association in other high risk populations, such as those of South-Asian, Turkish and Moroccan origin, is lacking [15, 19]. How ethnicity may affect this association seems particularly important as handgrip strength may differ significantly across ethnic groups [20]. Handgrip strength could, therefore, possibly explain ethnic differences in the prevalence of diabetes type 2. Evidence for this association could provide a lead for preventive interventions in high-risk populations or input for algorithms to identify people at risk of type 2 diabetes mellitus.

The aim of this study is therefore to investigate the association between handgrip strength and type 2 diabetes mellitus in a population-based sample of 18–70 year old men and women of six ethnic origins, living in Amsterdam the Netherlands. We will first describe the differences in handgrip strength between Dutch, South-Asian Surinamese, African Surinamese, Ghanaian, Turkish and Moroccan origin groups. Secondly, we will determine the association of handgrip strength with type 2 diabetes mellitus and whether this association is similar across ethnic groups. Finally, we will determine to what extent handgrip strength is associated with ethnic differences in type 2 diabetes mellitus.

## Materials and Methods

The current study is based on baseline data from the HELIUS (Healthy Life in an Urban Setting) study. The aims and design of the HELIUS study have been described before [21]. In brief, HELIUS is a large-scale prospective cohort study, which aims to unravel the causes of the unequal burden of disease among the largest ethnic groups in Amsterdam. The study started in 2011 and is carried out by the Academic Medical Center (AMC) Amsterdam and the Public Health Service of Amsterdam. Subjects were randomly sampled, stratified by ethnicity, from those aged 18–70 years listed in the Amsterdam Municipal Register [21, 22]. Data were collected by questionnaire and a physical examination, and biological samples were obtained during the physical examination. The study protocols were approved by the AMC Ethical Review Board, and all participants provided written informed consent.

### Study population

Baseline data collection is still on going. For the present analysis, we used the baseline data collected until June 2014, including 13316 participants for whom both questionnaire and physical examination data were available. Individuals with an unknown Surinamese origin or unknown/other ethnicity (n = 251) were excluded from our sample. Individuals with missing data on handgrip strength (n = 339) or type 2 diabetes mellitus (n = 74) were also excluded. Moreover, we excluded 58 people with a body fat percentage < 4% as this was considered a likely measurement error [23]. This resulted in a total study sample of 12.594 individuals: 2086 Dutch, 2216 South Asian Surinamese, 2084 African Surinamese, 1786 Ghanaian, 2223 Turkish and 2199 Moroccan origin participants.

### Measures and Definitions

#### Ethnicity

Ethnicity was defined according to the country of birth of the participant as well as that of his/her parents [21, 22]. Specifically, a participant is considered as of non-Dutch ethnic origin if he/she fulfils either of the following criteria: 1) he or she was born abroad and has at least one parent born abroad (first generation); or 2) he or she was born in the Netherlands but both his/her parents born abroad (second generation, [22]). The Surinamese group was further classified according to self-reported ethnic origin into ‘African’, ‘South Asian’, or other. Participants were considered of Dutch ethnicity if they and both parents were born in the Netherlands.

#### Type 2 diabetes mellitus

Prevalent type 2 diabetes mellitus was determined according self-reported physician diagnosis and/or or use of anti-diabetic medication and/or a fasting glucose level of ≥7.0 mmol/L (53 mmol/mol,[24]).

#### Handgrip Strength

Handgrip strength was assessed using a Citec handheld dynamometer (CIT Technics, Haren, the Netherlands). Subjects were seated in a chair without armrests and asked to sit up straight with their arms hanging loosely at their sides. On a verbal sign, subjects were asked to squeeze the handle as hard as possible. Two measurements of strength (in Newton) of both hands were taken with an interval of 1 minute. The highest of these four measurements was used in our analysis.

#### Other Factors

Data on body composition were obtained by measurement of height, body weight, hip circumference and waist circumference. These measurements were obtained in duplicate and the mean was used for analysis. However, a third measurement was taken if the difference between the two measurements was greater than 0.5 cm (height), 0.5 kg (weight), or 1.0 cm (hip and waist circumference). In that case the mean of the two measurements that were closest together was calculated. We calculated *BMI* as weight/height^2^, and waist-hip ratio as waist circumference/hip circumference. In addition, Body fat percentage (*BFP*) was assessed using arm-to-leg bioelectrical impedance analysis measuring impedance, resistance and reactance in Ohm at 50 Hz using a Bodystat 1500 analyzer (Bodystat Ltd, Isle of Man, UK). BFP was calculated using the proposed formula by Kyle et al [25].

*Education* was classified by the highest obtained educational degree in the Netherlands or the country of origin as: ‘No school’ (never been to school or elementary schooling only), ‘Lower education’ (lower vocational schooling or lower secondary schooling), ‘Intermediate education’ (intermediate vocational schooling or intermediate/higher secondary schooling) or ‘Higher education’ (higher vocational schooling or university).

*Smoking* was reported as current smoking or non-smoking.

*Alcohol use* was based on the first two questions of the Alcohol Use Disorders Identification Test (AUDIT): “How often do you drink alcoholic beverages?”, and “How many glasses of alcohol do you drink on a typical day that you drink?”. We used these two questions to classify alcohol use as ‘Abstinate’, ‘Moderate drinking’ (on average a maximum of 2 units alcohol a day, never more than 6 units a day) and ‘Excessive drinking’ (on average more than 2 units alcohol a day or more than 6 units a day).

*Physical activity score* was assessed with the Short Questionnaire to Assess Health-Enhancing Physical Activity (SQUASH) that has been validated in European-Dutch populations and has been used previously in multi-ethnic populations [26, 27]. The resulting SQUASH score (min/week) was divided into quartiles.

### Statistical analyses

The characteristics of our study population were reported as mean values with standard deviations for continuous variables and percentages for categorical variables. The mean handgrip strength was calculated separately for men and women [10–12, 14]. We used logistic regression to analyze the association between handgrip strength and type 2 diabetes mellitus, adjusted for socio-demographic variables (age, sex, ethnicity, height (as a proxy of childhood circumstances[28]) and education; Model 1). We report the association for every 10-point (in Newton) increase of handgrip strength. In Model 2 we further adjusted for BMI, percentage body fat, waist circumference and waist-hip ratio, hypothesizing that each variable discloses distinctive information on different aspects of body composition. We then added lifestyle factors (physical activity, smoking and alcohol use) in Model 3. In Model 4, we subsequently examined whether ethnicity modified the relationship between handgrip strength and type 2 diabetes mellitus by including an interaction term for ethnicity*handgrip strength into the model. The Likelihood Ratio test was used to assess interaction.

We performed several sensitivity analyses. First, we examined the consistency of the associations in subsets of the population in Models 5–8; men only, women only, to assess whether there would not only be a difference in mean handgrip strength but also a difference in association [10, 11, 13]. Normal weight (BMI = /< 25 kg/m2) or overweight (BMI > 25 kg/m2), to enable the comparison of findings with previous studies that have found association in specific subgroups (e.g. study among men [11, 12] or association among lean people only [14]). We also assessed interaction with Likelihood Ratio tests. Second, we repeated the analysis with type 2 diabetes mellitus defined by HbA1c in Model 9. Third, we repeated the analyses with newly identified diabetes, i.e. after excluding all people with self-reported type 2 diabetes mellitus hypothesizing that duration of type 2 diabetes mellitus could influence the association (Model 10).

Finally, we used logistic regression analysis to calculate the fully adjusted ethnic differences in type 2 diabetes mellitus. We examined to what extent handgrip strength explained ethnic differences by comparing the estimates of this model with the estimates of the model that additionally included handgrip strength. We considered a change of >10% in the odds ratio (OR) as potentially relevant.

All analyses were performed with SAS version 9.2 (SAS Institute Inc., Cary, NC, USA). A p value ≤ 0.05 was considered statistically significant.

The data underlying the analyses are available from the HELIUS study Executive Board. For details, we refer to http://www.heliusstudy.nl/nl/researchers/collaboration/.

## Results

The mean age was lowest among Moroccan participants and highest among the African Surinamese (Table 1). On average 42% of the participants were men. Education was highest in the Dutch origin population (60.2% attained higher education) and lowest in the Turkish population (34.2% reported no schooling). Body composition also varied across ethnic groups. BMI and waist circumference were highest in Turkish participants and BFP in Moroccan participants, while the Dutch participants had the lowest mean on all of these three variables. In all ethnic groups, the mean handgrip strength was higher for men than for women. Handgrip strength differed significantly between the ethnic groups (ANOVA p < 0.05). Among both sexes, handgrip strength was higher among Dutch than among the other ethnic groups. The lowest handgrip strength was found among South-Asian Surinamese men and women. Finally, we found that the prevalence of type 2 diabetes mellitus differed across the ethnic groups, ranging from 3.7% in the Dutch origin population to 19.5% in the South-Asian Surinamese (Table 1).

**Table 1:** Characteristics of the study population.

	Dutch		South Asian Surinamese		African Surinamese		Ghanaian		Turkish		Moroccan	
	N/mean	%/sd	N/mean	%/sd	N/mean	%/sd	N/mean	%/sd	N/mean	%/sd	N/mean	%/sd
**N =**	2086	16.1	2216	17.6	2084	16.5	1786	14.2	2223	17.7	2199	17.5
**Mean age (years)**	46.5	14.0	45.9	13.1	47.7	12.5	45.3	11.1	40.4	12.0	40.3	12.9
**Sex (men)**	960	46.0	991	44.7	754	36.2	730	40.9	1021	45.9	844	38.4
**Education** * **- No school**	62	3.0	352	16.0	148	7.2	529	30.2	755	34.2	732	33.6
**Education** * **- Lower**	302	14.6	745	33.8	716	34.6	675	38.5	544	24.7	396	18.2
**Education** * **-Intermediate**	462	22.3	628	28.5	726	35.1	439	25.0	606	27.4	699	32.1
**Education** * **- Higher**	1250	60.2	480	21.8	478	23.1	110	6.3	303	13.7	350	16.1
**Mean BMI (kg/m2)**	24.7	4.1	26.4	4.8	27.9	5.6	28.4	4.9	28.7	5.8	27.6	5.2
**Mean Waist circumference (cm)**	89.2	12.6	91.8	12.8	92.8	13.9	93.3	12.1	94.6	14.0	92.9	13.4
**Mean BFP**	29.0	7.4	32.1	8.3	32.5	8.8	32.5	8.9	32.2	8.3	32.9	8.5
**Mean WHR**	0.89	0.09	0.93	0.09	0.90	0.08	0.91	0.08	0.91	0.09	0.89	0.09
**Mean height (in cm)**	175.4	9.6	164.3	9.3	168.3	8.8	165.7	7.9	165.2	9.4	166.0	9.1
**Alcohol use** † **- Abstinate**	127	6.1	792	36	515	24.8	610	34.5	1504	68.2	1838	83.9
**Alcohol use** † **- Moderate**	1260	60.5	1302	59	1436	69.2	1067	60.4	672	30.5	344	15.7
**Alcohol use** † **- Excessive**	697	33.5	112	5	123	5.9	90	5.1	30	1.4	9	0.4
**Current smoking**	523	25.1	623	28	640	30.8	81	4.6	755	34.1	285	13.0
**Activity** ‡ **-Lowest quartile**	193	9.3	495	22	377	18.1	570	31.9	831	37.5	678	30.9
**Activity** ‡ **-25 to50** ^**th**^ **percentile**	495	23.7	584	26	521	25.0	397	22.2	544	24.5	603	27.5
**Activity** ‡ **-50 to75** ^**th**^ **percentile**	809	38.8	595	27	555	26.7	305	17.1	405	18.3	474	21.6
**Activity** ‡ **-Highest quartile**	588	28.2	539	24	628	30.2	513	28.7	438	19.8	442	20.1
**Mean HGS *Among men***	143.5	34.0	121.6	30.6	137.0	36.3	124.1	35.1	137.2	34.3	141.1	34.3
**Mean HGS *Among women***	87.6	21.6	68.0	18.6	80.0	22.9	76.8	23.6	76.8	22.4	78.5	20.8
**Type 2 diabetes mellitus** §	77	3.69	432	19.49	254	12.19	219	12.26	236	10.62	252	11.46

SD = standard deviation; BMI = body mass index; BFP = Body fat percentage measured with bioelectrical impedance [24]; WHR = waist-hip ratio; HGS = handgrip strength, the highest measurement of handgrip strength (in Newton)

* Highest obtained educational degree: ‘No school’ (never been to school or elementary schooling only), ‘Lower education’ (lower vocational schooling or lower secondary schooling), ‘Intermediate education’ (intermediate vocational schooling or intermediate/higher secondary schooling) or ‘Higher education’ (higher vocational schooling or university)

† Alcohol use based on the first two questions of the Alcohol Use Disorders Identification Test (AUDIT), classified as ‘Abstinate, ‘Moderate drinking’ (on average a maximum of 2 units alcohol a day, never more than 6 units a day) and ‘Excessive drinking’ (on average more than 2 units alcohol a day or more than 6 units a day)

‡ Physical activity score (min/week) assessed with the Short Questionnaire to Assess Health-Enhancing Physical Activity (SQUASH)

§Type 2 diabetes mellitus determined by a self-reported physician diagnosis, medication use or a fasting glucose level of ≥7.0 mmol/L (53 mmol/mol).

In all ethnic groups, handgrip strength was higher in the group without type 2 diabetes mellitus than in the group with type 2 diabetes mellitus (S1 Fig). After adjusting for socio-demographic factors, a ten point Newton increase in handgrip strength was associated with a 0.95 (95% CI: 0.93–0.97) lower odds of type 2 diabetes mellitus (Table 2, Model 1). Further adjustment for body composition and lifestyle factors only minimally changed this association (Models 2 and 3). Moreover, we did not find evidence for a difference in the association by ethnicity (Model 4).

**Table 2:** The association of maximum handgrip strength and type 2 diabetes mellitus.

		OR	95%-CI	
**Total population**	**Model 1:** Adjusted for age, sex, ethnicity, height and education	0.95	0.93	0.97
	**Model 2: Model 1 +** BMI, percentage bodyfat, waist circumference and waist-hip ratio	0.95	0.92	0.97
	**Model 3: Model 2 +** physical activity score, smoking and alcohol use	0.95	0.92	0.97
	**Model 4:** Model 3 + interaction by ethnicity			
	*Dutch*	1		*
	*South Asian Surinamese*	0.97	0.90	1.04
	*African Surinamese*	0.95	0.88	1.02
	*Ghanaian*	1.01	0.93	1.09
	*Turkish*	0.96	0.89	1.04
	*Moroccan*	0.97	0.90	1.05
**Within subset of population**	**Model 5:** Model 3 in Men	0.98	0.95	1.01*
	**Model 6:** Model 3 in Women	0.91	0.87	0.95
	**Model 7:** Model 3 in BMI ≤ 25 kg/m2	0.96	0.90	1.02*
	**Model 8:** Model 3 in BMI > 25 kg/m2	0.94	0.92	0.97
**With alternative outcomes**	**Model 9:** Model 3 with HbA1c-defined type 2 diabetes mellitus†	0.97	0.94	1.99
	**Model 10:** Model 3 with Newly identified type 2 diabetes mellitus only‡	0.99	0.94	1.04

BMI = body mass index; OR = odds ratio given for every 10 Newton increase in handgrip strength, with type 2 diabetes mellitus determined by a self-reported physician diagnosis, medication use or a fasting glucose level of ≥7.0 mmol/L (53 mmol/mol); CI = confidence interval; p-value: p value of the likelihood ratio test for handgrip strength

* p-value of the likelihood ratio test for the interaction by ethnicity was non-significant, by sex p = 0.0269 and by BMI (continuous) p = 0.6645. For sex and BMI the stratified analyses are shown in the table.

† HbA1c-defined type 2 diabetes mellitus

‡Participants with self-reported diabetes excluded.

The estimated association between handgrip strength and type 2 diabetes mellitus did not differ substantially between men and women (Table 2), despite the interaction being significant (p = 0.0269). The association also appeared similar when we studied only overweight or only normal weight subjects (Models 7 and 8; interaction BMI p = 0.6645). Moreover, the analyses with HbA1c defined type 2 diabetes mellitus were in line with the analysis based on fasting plasma glucose (Model 9). However, exclusion of all people with known type 2 diabetes mellitus attenuated the estimated association between handgrip strength and type 2 diabetes mellitus (OR 0.99, 95% CI: 0.94–1.04; Table 2, Model 10).

Finally, ethnic differences in type 2 diabetes mellitus were not explained by adjustment for handgrip strength (Table 3). For instance, the odds ratio for South-Asian Surinamese versus the Dutch changed from 5.03 (95% CI 3.69–6.86) in the fully adjusted model to 4.87 (95% CI 3.57–6.65) after adjustment for handgrip strength. The changes in the association for the other ethnicities showed a similar pattern.

**Table 3:** Ethnic differences in type 2 diabetes mellitus, adjusted for known risk factors and handgrip strength.

	Fully adjusted*			Fully adjusted* + handgrip strength		
	OR	95%-CI		OR	95%-CI	
**Dutch**	1.00			1.00		
**South Asian Surinamese**	5.03	3.69	6.86	4.87	3.57	6.65
**African Surinamese**	2.72	2.01	3.68	2.69	1.99	3.65
**Ghanaian**	2.92	2.11	4.05	2.82	2.03	3.91
**Turkish**	2.57	1.81	3.66	2.58	1.81	3.67
**Moroccan**	3.29	2.33	4.65	3.35	2.37	4.72

OR = odds ratio for type 2 diabetes mellitus determined by a self-reported physician diagnosis, medication use, or a fasting glucose level of ≥7.0 mmol/L (53 mmol/mol); CI = confidence interval

*Adjusted for age, sex, ethnicity, height and education, BMI, waist circumference, waist-hip ratio, percentage body fat, physical activity score, smoking and alcohol use.

## Discussion

The ethnic differences in hand grip strength in our study and the consistent finding of an association with type 2 diabetes mellitus among all ethnic groups, among both sexes, and among lean and overweight individuals suggests that handgrip strength may be a relevant marker to investigate further among high-risk populations. For instance, since handgrip strength is a non-invasive, low-cost measure, it may be a useful marker to help identify people at risk of type 2 diabetes mellitus in clinical or public health practice. Moreover, if the consistency and causality of the association is confirmed, muscle mass and muscle strength may potentially be a target for risk reduction in multi-ethnic populations at high-risk of type 2 diabetes mellitus through focused training [16]. We focused only on the static measure of handgrip strength. The dynamic measure involving training and improvement of muscle mass and muscle quality was not part of our study, but shows enhancement of insulin sensitivity and improvement of body fat composition [16].

However, the value of strategies for risk stratification or the prevention of type 2 diabetes mellitus in multi-ethnic populations remains to be determined. Importantly, our results also show that a focus on handgrip strength alone may not reduce the disparities between groups.

We found that the mean handgrip strength varied widely across ethnic groups. A greater handgrip strength was associated with a lower prevalence of type 2 diabetes mellitus in all ethnic groups with no difference in association across the ethnic groups. Further analyses within subsets of the population did not significantly change our results. However, exclusion of people with known type 2 diabetes mellitus did attenuate the association. Finally, despite ethnic differences in handgrip strength and an association of handgrip strength with type 2 diabetes mellitus, we found that these differences in handgrip strength did not explain the differences in the prevalence of type 2 diabetes mellitus across ethnic groups.

Our finding of large differences in handgrip strength between ethnic groups seemed in line with a previous study [20]. However, while in our study particularly South-Asian Surinamese men and women had a low handgrip strength as compared to their Dutch counterparts, previous work only included people of Hispanic, African American and European origin. Moreover, African Americans had a higher handgrip strength compared to people of European origin, while in our study the groups of African origin (African Surinamese and Ghanaian) did not have a greater handgrip strength than their Dutch counterparts. The differences between ethnic groups and between studies may not only reflect differences predisposition, but also differences in environmental or lifestyle influences (e.g. through epigenetics [29]).

Despite the differences between ethnic groups, the differences between men and women within the groups were consistent in both studies and also in line with previous studies that showed large variations between the two sexes [10, 11, 13].

The association between maximum handgrip strength and type 2 diabetes mellitus among different ethnic groups living in the same country has not been studied extensively. However, the negative association in our multiethnic population seems in line with the recently reported association of the maximum HGS with incident diabetes in the cross-country comparative PURE study [30]. The negative association of handgrip strength and type 2 diabetes mellitus among both men and women in our study seems consistent with the finding in previous studies that muscle quality is lower in men and women with diabetes than those without diabetes, and that lower grip strength is associated with a higher cardiovascular mortality [10, 11, 13–15, 19]. Our findings also are also consistent with a prospective study among Japanese Americans that reported an association between a higher handgrip strength and a lower risk of developing type 2 diabetes mellitus over the course of 10 years, in particular among lean individuals [14]. In contrast to that study, we did not find evidence for a different association among normal weight as compared with overweight individuals. We hypothesize that this may be because, in the long term, the enduring negative effect of overweight overshadows the potential benefit of a great handgrip strength, particularly showing in new onset of type 2 diabetes mellitus that the Japanese study focused on, and less in the prevalence that our study focused on.

One important limitation of our study is that, due to the cross sectional design of our study, we were not able to study the temporal association between handgrip strength and new onset of type 2 diabetes mellitus as was done in previous studies [14, 30]. However, we did evaluate whether exclusion of those with known type 2 diabetes mellitus affected the analyses. Although the effect on the reported association may simply reflect an effect on the power of the analyses, it could also imply a lack of a causal relation in our population. Those with known type 2 diabetes mellitus were arguably the cases with longer diabetes duration. Park et al. found that a longer duration of diabetes or poor glycemic control were with poorer muscle quality [11]. In addition to being insulin insensitive, excess visceral adipose tissue up-regulates the inflammatory response [31, 32] which may lead to catabolism and may consequently contribute to further decline in muscle mass and quality. This emphasizes the importance of further work in multi-ethnic populations living under similar circumstances with more specific, mechanistic analyses in a longitudinal setting.

A second limitation of our results is the measurements used in our study. We used a single measurement of fasting plasma glucose and HbA1c to define type 2 diabetes mellitus. Although our definition was based on the WHO criteria and is in line with previous epidemiological studies, we may have missed cases that would have been identified if an oral glucose tolerance test had been used [24, 33]. Because the overlap between measures may vary between ethnic groups the relative differences between groups may have been attenuated [34, 35].

Furthermore, several of the measures were (partially) defined based on self-reported data. Self-reported data are subject to recall bias and may be affected by social desirability. For instance, the self-reported physical activity may not fully capture differences in activity patterns, duration and intensity [27]. This may be relevant as exercise is associated with a greater handgrip strength and at the same time improves glycemic control and lowers visceral adipose tissue [36]. Additionally, as in previous epidemiological studies, we were not able to include measures reflecting the different types of fat, e.g. the amount of visceral and subcutaneous adipose tissue. Several more complex methods are necessary to measure these, such as magnetic resonance images, computed tomography, and dual-energy X-ray absorptiometry [37–40]. This omission may have led to incomplete adjustment and to overestimation of the associations in the study.

## Conclusions

In conclusion, we found large ethnic differences in handgrip strength, a marker of muscle strength and muscle quality. A lower handgrip strength was consistently associated with a higher prevalence of type 2 diabetes mellitus in all ethnic groups, but handgrip strength did not explain ethnic differences in the prevalence of type 2 diabetes mellitus. If these findings are confirmed in prospective studies among multi-ethnic populations, muscle strength and quality may be considered as a possible additional target for preventive intervention or handgrip strength as a marker to help identify people at risk of type 2 diabetes mellitus in clinical or public health practice. Clearly, the value of such strategies should be evaluated.

## Supporting Information

S1 Fig Mean handgrip strength (in Newton) among people with or without type 2 diabetes mellitus in the different ethnic groups, adjusted for age and sex. Errorbars indicate the 95%-confidence interval. HGS = handgrip strength, the highest of four measurements of handgrip strength (in Newton); Type 2 diabetes determined by a selfreported physician diagnosis, medication use or a fasting glucose level of ≥7.0 mmol/L (53 mmol/mol); p< 0.05 differences between people with and without type 2 diabetes in all groups.
(TIFF)
