# Supplementary figures and images for: The Association of Handgrip Strength and Type 2 Diabetes Mellitus in Six Ethnic Groups: An Analysis of the HELIUS Study
Source: PLoS One. 2015 Sep 14;10(9):e0137739. doi: 10.1371/journal.pone.0137739 (PMC4569584; doi:10.1371/journal.pone.0137739)

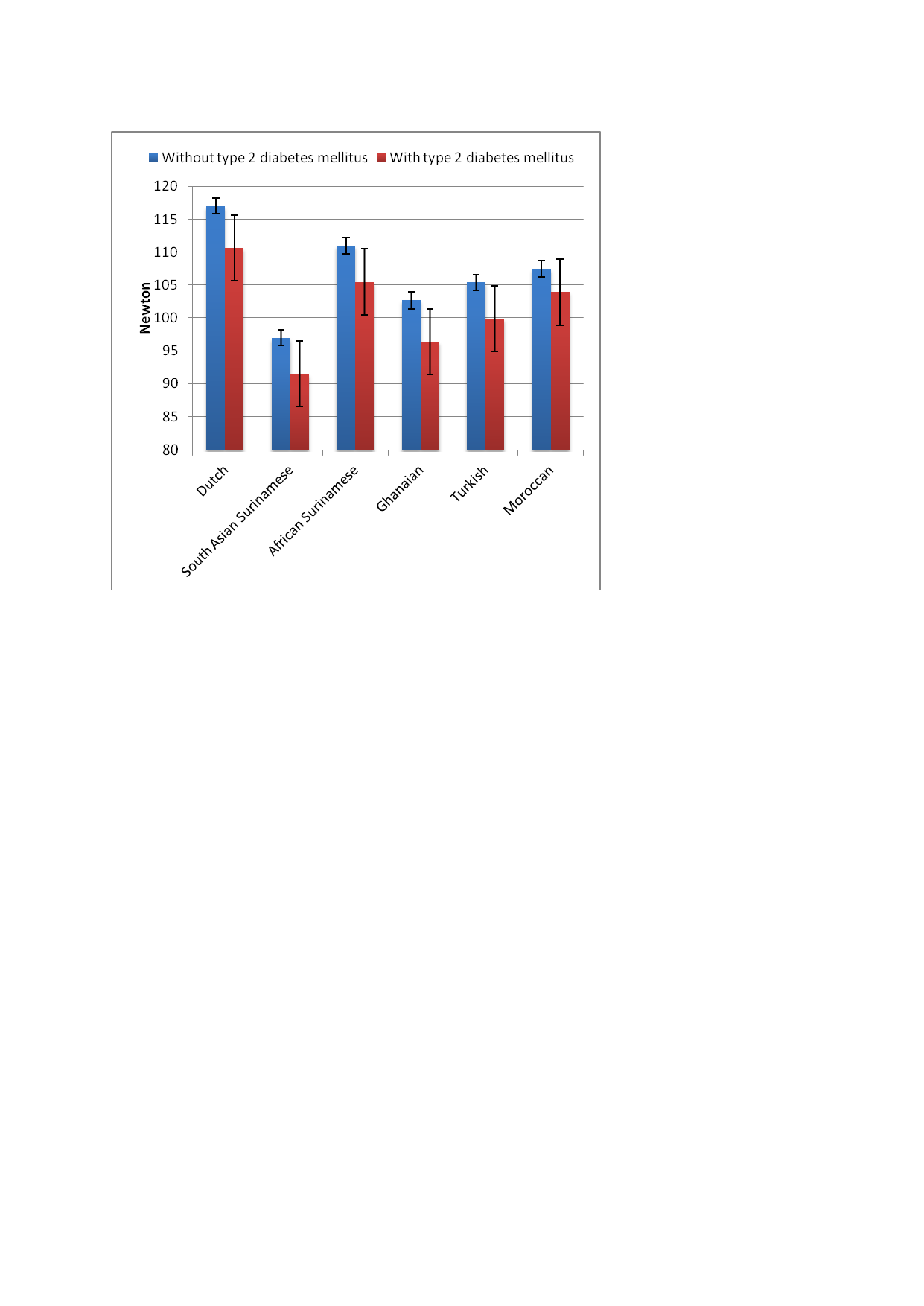

Supplement: S1 Fig — Errorbars indicate the 95%-confidence interval. HGS = handgrip strength, the highest of four measurements of handgrip strength (in Newton); Type 2 diabetes determined by a selfreported physician diagnosis, medication use or a fasting glucose level of ≥7.0 mmol/L (53 mmol/mol); p< 0.05 differences between people with and without type 2 diabetes in all groups. (TIFF) [file pone.0137739.s001.tiff]
